# New insights into the food web of an Australian tropical river to inform water resource management

**DOI:** 10.1038/s41598-020-71331-0

**Published:** 2020-08-31

**Authors:** Leah S. Beesley, Bradley J. Pusey, Michael M. Douglas, Daniel C. Gwinn, Caroline A. Canham, Chris S. Keogh, Oliver P. Pratt, Mark J. Kennard, Samantha A. Setterfield

**Affiliations:** 1 School of Biological Sciences, The University of Western Australia, Perth, 6009 Australia; 2 Research Institute for Environment and Livelihoods, Charles Darwin University, Darwin, 0909 Australia; 3 Biometric Research, South Fremantle, 6162 Australia; 4 Australian Rivers Institute, Griffith University, Brisbane, Australia; 5 Northern Australia Environmental Resources Hub, National Environmental Science Program, Casuarina, Australia

**Keywords:** Freshwater ecology, Stable isotope analysis

## Abstract

Rivers around the world are threatened by altered flow due to water resource development. Altered flow can change food webs and impact riverine energetics. The Fitzroy River, in northern Australia, is targeted for development but uncertainty remains about the sources of carbon supporting the food web, particularly in the lowlands—the region most likely to be impacted by water extraction. This study used stable isotopes to investigate if algal biofilm is the main carbon source sustaining fish in lowland habitats. We also sought evidence that large-bodied migratory fish were transporting remote carbon around the system. Our results revealed that local algal biofilm carbon was the dominant source of energy sustaining fish in wet season floodplain habitats, but that fish in main-channel pools during the dry season were increasingly dependent on other carbon sources, such as leaf litter or phytoplankton. We found no evidence that large-bodied fish were transporting remote carbon from the floodplain or estuary into the lower main-channel of the river. We recommend that water planners take a precautionary approach to policy until sufficient food web evidence is amassed.

## Introduction

Freshwater ecosystems sustain remarkable biodiversity and provide important services to nature and society but are degrading globally at an increasing rate^1^. A common threat to freshwater systems is changes in flow-regime arising from water resource development to support agriculture^2,3^. Changes in flow regime can alter hydrological connectivity between the river and its estuary, the river and its floodplain, and between surface and subsurface water^2,4,5^. Altered connectivity may reduce the movement of nutrients and animals, and change the flow of energy through the food web, ultimately reducing the abundance of species valued by people (e.g. fish)^6,7^. Understanding how riverine food webs function is essential if we are to estimate the likely impacts of flow alteration, make informed water management decisions and enact effective policy. The implementation of environmental flows policy is now considered critical to reverse the decline of freshwater biodiversity^8^.

There is an increasing focus on water management decisions and environmental flow policy in the wet-dry tropics of northern Australia, as pressure mounts to develop the region’s large river systems^9,10^. The region includes some of Australia’s most intact freshwater ecosystems, which support high biodiversity, numerous endemic species and many fish which move between riverine, floodplain and estuarine environs^11^. The Fitzroy River, in the Kimberley region of north-western Australia, has been identified as having potential for development^10^, and at least one application to divert water from the river to grow fodder crops for cattle is being considered by government. This intermittent river, 700 km in length^10^, is highly valued by the Indigenous peoples who live along it, and for whom customary harvest of large-bodied fish and freshwater prawns is important^12^. Water managers are tasked with setting water allocation policies that protect the ecological and cultural values of the river, particularly those of the river’s lower reaches that are most likely to be impacted by water extraction. However, there are scant ecological data from the Fitzroy River to support decision making^13^, but see^14^ for an exception.

A key knowledge gap for the Fitzroy River is the extent to which the energetic function of the riverine ecosystem relies on various aspects of the flow regime^13^. Food web studies that use stable isotopes to trace the flow of energy (carbon) through the food web are useful for understanding riverine energetics because they can identify the sources (e.g. algae, riparian leaves, phytoplankton) and locations (e.g. main-channel, floodplain, estuary) of energy that underpin consumer production^15^. Previous food web research undertaken in the upper reaches and tributaries of the Fitzroy River found that isotopic ratios of carbon ^13^C/^12^C in dry season pools were closely coupled with values of local algal biofilm, indicating that this was the dominant energy source sustaining fish production^16,17^. This suggests that floodplains do not deliver an important energetic subsidy to the river, in contrast to other tropical rivers in Australia^18–20^ and elsewhere^21,22^. The Fitzroy River’s floodplain may play a minimal energetic role because it is relatively small and only inundated briefly^16^. High dependence on local algae also suggests that riparian vegetation plays a limited role in the aquatic food web in the Fitzroy River. If representative of the broader river, these findings lessen the policy imperative to protect flows that inundate the floodplain and sustain riparian trees to maintain existing food web dynamics. However, uncertainty remains regarding the spatial generality of these findings because they were conducted in reaches upstream from where the impacts of water extraction are predicted to occur (i.e. upper reaches and tributaries *vs* lowland reaches respectively). If the findings of previous studies are not broadly applicable, water policy may fail to protect key flow components that maintain primary production and higher order consumers (e.g. fish and prawns).

Several factors suggest the findings of previous upland food web studies may not necessarily be applicable to the lower Fitzroy River. First, upland sites have a shorter duration of connection to the floodplain because steeper topography and lower water volumes reduce the magnitude and duration of inundation compared to lowland sites^23^. Second, upland sites typically have a cobble or gravel substrate which is ideal for benthic algae growth but is not representative of the lowland river which is sandy. Production of benthic algae and its incorporation into the food web may be considerably lower in sandy reaches, particularly during periods of high flow^24^. Third, previous studies were conducted after relatively small wet seasons which should minimise the apparent energetic importance of the floodplain and the estuary^25,26^. Finally, these prior studies included relatively few fish species. For example ^16^ only examined nine fish species in a river that supports 23 species of freshwater fish^27^, and^17^ examined even fewer. Importantly, previous studies did not include two abundant large-bodied migratory fish, *Lates calcarifer* (barramundi) and *Neoarius graeffei* (fork-tailed catfish). These species are valued by indigenous peoples^12^ and have been shown elsewhere in northern Australia to link river-floodplain-estuary food webs through space and time^28^. Furthermore, barramundi is a highly valued target of recreational and commercial fishers.

This study aimed to improve our knowledge of food webs in floodplain and main-channel habitats of the lower Fitzroy River, to better understand the potential impacts of proposed water extraction and to inform management policies. We sought to better understand the sources of carbon underpinning fish production. We focused on two main questions, (1) is algal biofilm the main carbon source supporting food webs in the lowlands of the river? and (2) is there evidence that large-bodied migratory fish are transporting carbon around the system? We analysed data sets collected in the lower river that contained 17 of the 23 known fish species in the river, including *Lates calcarifer*, *Neoarius graeffei,* and *Macrobrachium spinipes* (freshwater prawn). We hypothesised that algal biofilm would be the dominant basal carbon source for fish sampled on the floodplain in the wet season. In contrast, we expected that fish and prawns in dry-season, main river channel pools would rely more on non-algal biofilm sources, e.g. leaf litter and/or phytoplankton. We also hypothesised that large-bodied migratory species would show evidence of carbon from remote sources, i.e. from the floodplain or estuary (as per^28^). The outcomes of this research increase the scientific evidence available to support water resource management for this data-poor river system.

## Results

**Figure 1:**
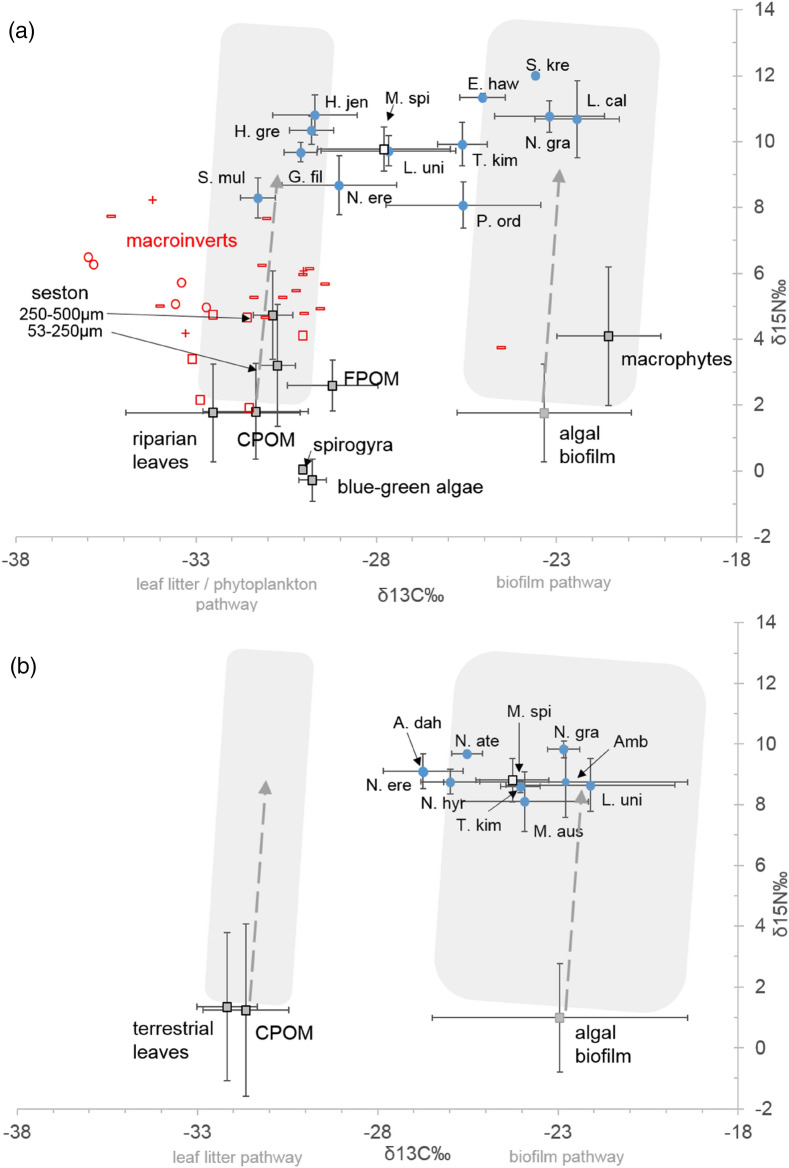
Stable isotope biplots (δ^13^C and δ^15^N) showing the food web of lowland habitats in the Fitzroy River; showing (**a**) main-channel pools during the dry season and (**b**) floodplain habitats during the wet season. Data were pooled across sites for each time period, and fish muscle with C:N ratios > 3.5 was corrected for lipid. Mean (± SD) isotope values are provided for local carbon sources (grey-filled squares), fish (blue-filled circles) and the freshwater prawn (white-filled square). Individual values are shown for macroinvertebrate functional feeding groups (red symbols, where ‘open square’ denotes scrapers, ‘open circle’ denotes filterers, ‘minus’ denotes predators and ‘plus’ denotes multiple functional groups). Fish species codes are A. dah = *Anodontiglanis dahli*, Amb = Ambassis spp., E. haw = *Elops hawaiensis*, G. apr = *Glossamia aprion*, G. fil = *Gerres filamentosus*, H. gre = *Hannia greenwayi*, H. jen = *Hephaestus jenkinsi*, L. cal = *Lates calcarifer*, L. uni = *Leiopotherapon unicolor*, M. aus = *Melanotaenia australis*, M. spi = *Macrobrachium spinipes* (freshwater prawn), N. ere = *Nematalosa erebi*, N. ate = *Neosilurus ater,* N. gra = *Neoarius graeffei*, N. hyr = *Neosilurus hyrtlii*, P. ord = *Planiliza ordensis*, S. mul = *Selenotoca multifasciata*, S. kre = *Strongylura krefftii*, and T. kim = *Toxotes kimberleyensis*. Grey filled boxes show the two alternate carbon pathways, an algal biofilm pathway and a non-algal biofilm pathway, i.e. a leaf litter and/or phytoplankton pathway. Dashed arrows in the boxes represent changes in the mean δ^13^C values of the two alternate source pathways as they enrich up the food web and the margin of the boxes encompass 1 s.d. of the mean.

A total of 268 samples were analysed. Samples from lowland main-channel pools included six carbon sources, 17 invertebrate families, including the freshwater prawn, and 12 fish species. δ^13^C values of sources overlapped considerably (Fig. 1a). Leaf litter and the surrogate of phytoplankton (i.e. 53–250 µm fraction of seston) were highly depleted in ^13^C, with mean δ^13^C values of − 32.3 and − 30.8 ‰, respectively (Fig. 1a, Supplement Table S1). Algal biofilm was considerably more enriched, with a mean δ^13^C value of -23.3 ‰ (Fig. 1a, Supplement Table S1). Most macroinvertebrates exhibited highly negative δ^13^C values between − 30 to − 33 ‰, suggesting they obtained their energy from terrestrial carbon sources and/or phytoplankton (Fig. 1a, Supplement Table S2). Fish in main-channel habitats displayed considerable interspecific variation in δ^13^C values, and variation in δ^15^N values, indicating dietary partitioning and use of multiple carbon sources (Fig. 1a, Supplementary Table S3). A subset of sources and consumers were collected on the floodplain. Floodplain leaf litter was highly depleted in ^13^C (mean δ^13^C − 31.9‰) and algal biofilm was relatively enriched (mean − 22.9‰) (see Fig. 1b, Table 1); similar to values observed within the main river channel. A total of nine small-bodied fish species and the freshwater prawn were collected on the floodplain (Supplement Table S3).

**Table 1:** Source estimates used in the mixSIAR mixing model for the main-channel and the floodplain data sets.

Basal source	n	δ^13^C		δ^15^N	
		Mean	St dev	Mean	St dev
**Main-channel dataset (Oct 2017)**					
Algal biofilm	4	− 23.3	2.42	1.77	1.48
CPOM + seston 53–250 μm	8	− 31.1	1.07	2.73	1.80
**Floodplain dataset (Mar 2018)**					
Algal biofilm	5	− 22.9	3.55	0.99	1.78
CPOM	4	− 31.9	0.89	1.30	2.15

CPOM refers to coarse particulate organic matter (i.e. leaf litter).

### What is the main carbon source supporting food webs in the lowlands of the river?

All mixing models exhibited convergence (i.e. Gelman–Rubin statistic < 1.01 for all models). Model selection using LOOic revealed that fish dietary isotope values (δ^13^C, δ^15^N) were generally best predicted when mixing models accounted for variation in isotope proportions among species. This was true for herbivores and omnivores in main-channel and floodplain habitats as indicated by high model probabilities for models containing the species effects (Table 2, note the exception of floodplain herbivores which were only represented by a single species). Alternatively, we found little evidence of variation in isotope proportions among carnivore species in the main-channel; however, no single model was highly probable indicating high model uncertainty for this trophic guild (Table 2). We also found that isotope proportions varied among individual lengths within species and sample sites, but these effects were inconsistent among trophic guilds (Table 2). For example, isotope proportions were best predicted by a model accounting for variation among sites for main-channel herbivores (Model 5, Table 2), while isotope proportion were best predicted by a model accounting for variation among individual lengths for main-channel omnivores (Model 7, Table 2). Model-averaged estimates for length indicated that the proportion of algal biofilm carbon tended to increase with fish length. This effect was significant for both main-channel omnivores (effect size omnivores = 0.487, [95% CI 0.256, 0.732]) and floodplain omnivores (effect size omnivores = 0.091, [95% CI 0.010, 0.177]). Changes in diet with length were not significant for other trophic guilds and habitat combinations, i.e. 95% CI’s overlapped zero.

**Table 2:** Model subsets investigating the proportion of local algal biofilm in fish diets for fish in main-channel pools and floodplain habitats.

Model subsets	Main-channel			Floodplain	
	Herbivores	Omnivores	Carnivores	Herbivores	Omnivores
(1) NULL	0.000	0.000	0.089	**0.423**	0.000
(2) Site	0.000	0.000	0.127	0.157	0.000
(3) Species	0.001	0.000	0.104	–	0.000
(4) Length	0.000	0.000	**0.243**	0.366	0.000
(5) Site + species	**0.907**	0.000	0.109	–	0.050
(6) Site + length	0.000	0.000	0.109	0.054	0.000
(7) Species + length	0.001	**0.832**	0.099	–	0.000
(8) Site + species + length	0.091	0.168	0.121	–	**0.950**

Model support is shown using model weights (Akaike weights) and describes the probability that the model will make the best predictions on new data given the set of candidate models^29^. Model weights are based on LOOic—the leave-one-out cross-validation information criterion using Bayesian posterior model probabilities. Models were run using the mixing model mixSIAR (Stock et al.^30^) and were run separately for herbivorous, omnivorous and carnivorous fish to allow different trophic enrichment inputs for these different groups. Note, only one herbivore was present in the floodplain dataset, hence models with species were irrelevant; no carnivores were collected on the floodplain.

**Figure 2:**
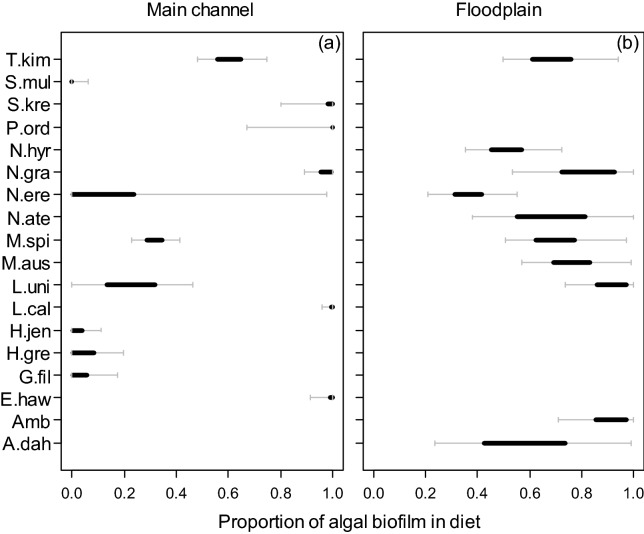
Isotope mixing-model (mixSIAR) estimated proportion of algal biofilm to the diet (muscle tissue) of fish collected from (**a**) main-channel sites during the late dry season (October 2017) and (**b**) floodplain site during the wet season (March 2018). Low proportions of algal biofilm refer to high proportions of the alternate source, which is riparian leaf litter/phytoplankton for (**a**) and leaf litter for (**b**). Plots show means, 50% (bolded black) and 95% Bayesian credible intervals. Fish species codes are as per Fig. 1.

Model-averaged estimates of isotope proportions for species revealed that fish inhabiting the wet season floodplain were supported predominantly by local algal biofilm carbon, whereas fish in dry season main-channel pools were supported by a mix of algal biofilms and leaf litter and/or phytoplankton. For instance, more than half (60%, n = 6) of fish species collected on the floodplain obtained more than 50% of their dietary carbon from algal biofilms, i.e. 95% Bayesian credible intervals were ≥ 0.5 (Fig. 2b, Supplementary Table S4). These species included *Leiopotherapon unicolor*, *Ambassis* spp., *Melanotaenia australis*, *N. graeffei*, *Toxotes kimberleyensis* and the freshwater prawn *M. spinipes*. All species but two, the herbivore/detritivore *N. erebi*, and *Anodontiglanis dahli*, obtained more than 30% of their energy from algal biofilms (Fig. 2b, Supplementary Table S4).

In contrast, in main-channel pools during the dry, only one third (38%, n = 5) of species obtained more than 50% of their energy from algal biofilms (i.e. 95% Bayesian credible intervals were ≥ 0.5, Fig. 2a, Supplementary Table S5). This included the two large-bodied migratory species, *N. graeffei* and *L. calcarifer*, the mullet *Planiliza ordensis*, and the other carnivorous species *Elops hawaiensis*, and *Strongylura krefftii* (Fig. 2a). Nearly half of all species surveyed (46%, n = 6) obtained the majority of their dietary carbon from leaf litter or phytoplankton sources. This included three terapontid grunter species, *Hephaestus jenkinsi*, *Hannia greenwayi* and *L. unicolor*, two estuarine species *Selenotoca multifasciata* and *Gerres filamentosus*, as well as the freshwater prawn *M. spinipes* (Fig. 2a). The model displayed uncertainty about the diet of bony bream, *N. erebi*, although the 50th percentiles suggested a strong reliance on leaf litter or phytoplankton, the 95% percentiles included the possibility of algal biofilm contributing to the diet (Fig. 2a).

### Is there evidence that large-bodied migratory fish are transporting carbon around the system?

**Figure 3:**
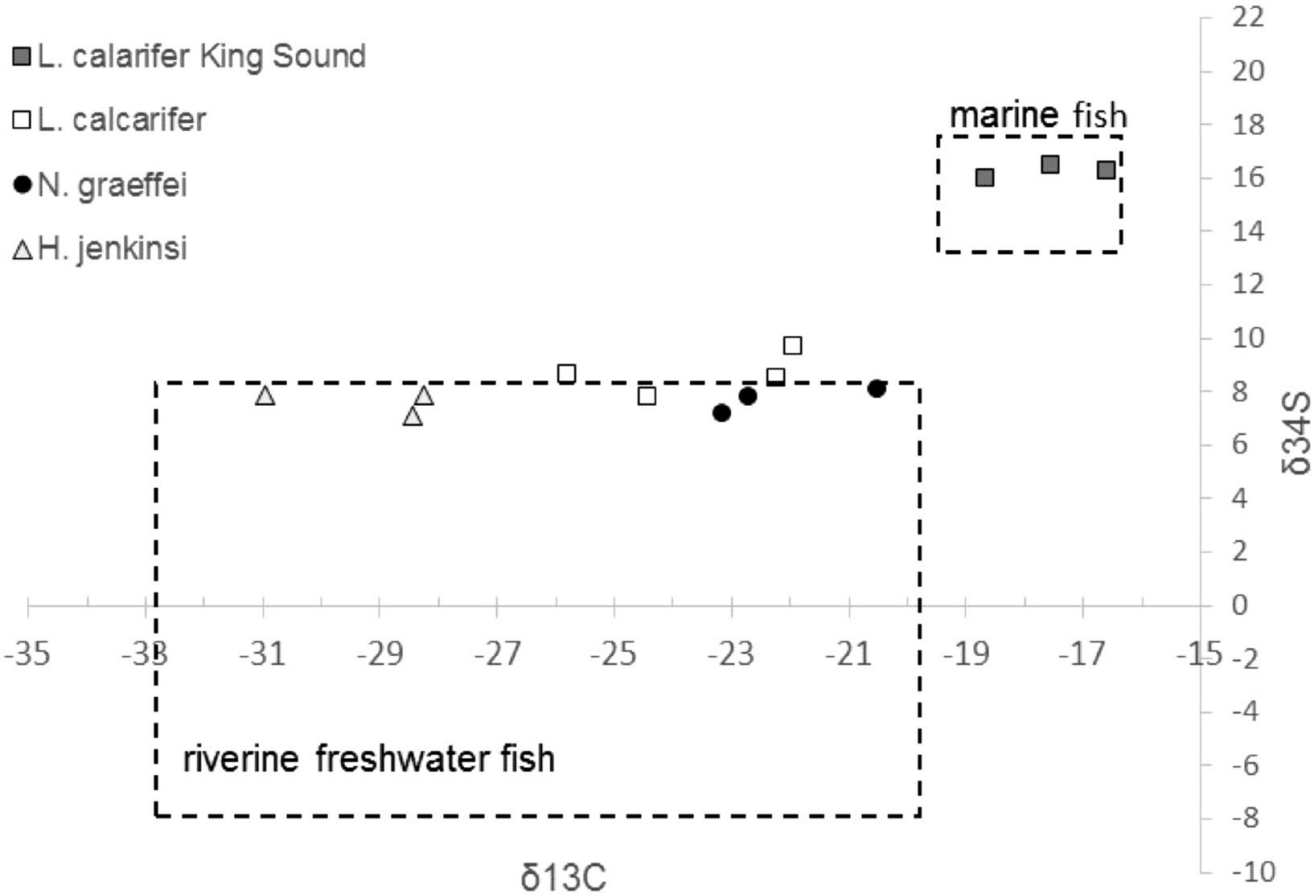
Stable isotope biplot (δ^13^C and δ^34^S) for large-bodied migratory (*L. calcarifer* and *N. graeffei*) and non-migratory (*H. jenkinsi*) fish in main-channel pools of the lower Fitzroy River during the dry season (October 2017). *L. calcarifer* collected opportunistically from the coastal environment into which the river debouches (King Sound) are also shown. Dashed rectangles describe typical δ^34^S values for marine and freshwater fish, from − 8 to + 8 as per^31^.

We found little evidence that large-bodied fish contained remote carbon. However, our ability to investigate an energy subsidy from the floodplain to the main-channel was hampered by overlapping algal biofilm δ^13^C values in these two habitats. For instance, a Kruskal–Wallis rank sum test revealed that δ^13^C values for algal biofilm were similar in floodplain and main-channel habitats (Kruskal–Wallis χ^2^ = 0.240, df = 1, p = 0.624). Isotopes of sulphur differed markedly between fish from the marine and freshwater environment with *L. calcarifer* caught in the estuary into which the river debouches (King Sound) displaying enriched δ^34^S values compared to freshwater fish (Fig. 3). While the divergence in δ^34^S between freshwater and estuarine fish enabled, in theory, an assessment of estuarine-freshwater subsidies, there was no evidence that large-bodied migratory species (*L. calcarifer*, *N. graeffei*) caught in main-channel pools contained a marine subsidy. That is, these fish displayed δ^34^S values typical of freshwater (between − 8 to + 8 ‰^31^, Fig. 3), were statistically similar to the non-migratory control species *H. jenkinsi* (Kruskal–Wallis χ^2^ = 5.432, df = 2, p = 0.066), and were statistically distinct from fish collected in the estuary (Kruskal–Wallis χ^2^ = 6.464, df = 1, p = 0.011).

## Discussion

Our study provided new insights about the food web in the lowlands of the Fitzroy River. We found that local algal biofilm carbon was the dominant source of energy sustaining fish on the floodplain during the wet season, but that fish and prawns in lowland pools during the dry season were increasingly dependent on other carbon sources, such as leaf litter or phytoplankton. We found no evidence that large-bodied fish were transporting remote carbon from the floodplain or estuary into the river. Our results highlight the risk associated with making food web generalisations across a river system. We recommend that water planning policy takes a precautionary approach until sufficient food web evidence is amassed from a range of habitat and flow contexts in the lower section of the Fitzroy River—the region most likely to be impacted by water resource development.

The dominant source of energy sustaining fish assemblages varied between lowland habitats. Fish inhabiting the wet season floodplain relied on energy from algal biofilms, consistent with earlier findings in the Fitzroy, and other systems in northern Australia (see review by^32^ and^16,20^), and is likely due to the high nutritional value of algal biofilms relative to leaf litter^33,34^. In contrast, fish inhabiting dry season main-channel pools relied predominantly on non-algal biofilm sources of energy, such as leaf litter or phytoplankton. Although regionally atypical^35^, an important energetic role for leaf litter detritus and zooplankton has been found in other lowland river habitats in Australia^7,36,37^. In the lower Fitzroy River, the species utilising the most non-algal biofilm energy included terapontid grunters, *N. erebi* and *Macrobrachium* sp. These species have been found in the Fitzroy and elsewhere to either consume large amounts of detritus^7,36,38^ or display trophic flexibility^38,39^, both of which were observed in our study.

We found no direct evidence that large-bodied migratory fish in lowland main-channel pools (i.e., *L. calcarifer* and *N. graeffei*) were sustained by remote carbon, from either the floodplain or the estuary. This finding contrasted with our expectation based on results from other rivers of northern Australia^20,28,40^. This finding may reflect a fundamental difference in the ecology of the Fitzroy River system; however, it may also be attributed to limitations in our data sets. For example, our ability to investigate floodplain subsidies was hampered by overlapping algal biofilm δ^13^C and δ^15^N values in the main-channel and the floodplain, a common problem when using stable isotopes^41^. Furthermore, the absence of marine carbon in fish sampled in the lower Fitzroy River may be because of low replication and because fish were collected 6 months after the wet season. *L. calcarifer*, particularly young-of-year fish, are known to grow rapidly within freshwater^42^, and the addition of new tissue during this 6-month period may have masked any marine signal. We recommend future research targets young-of-year *L. calcarifer*, recently emigrated upstream from the estuary, to better assess the extent of marine subsidy.

Although we could not conclusively identify an energetic role for floodplain carbon for fish in the main-channel of the Fitzroy River, several observations suggest it exists. For example, we observed many young-of-the-year fish using the floodplain. It is likely these fish retreated to the main-channel of the river as the floodplain contracted during the dry season, as is common in many rivers around the world^43,44^ taking newly-assimilated carbon with them. Furthermore, Indigenous peoples have observed that *L. calcarifer* occupy areas where flood-runner creeks join the main-channel and feed on small-bodied fish returning from the floodplain^45^. Elsewhere in northern Australia, *N. graeffei* and *L. calcarifer* have been shown to link riverine food webs in time and space^28^. Radiotelemetry research in another northern Australian river revealed that during flooding, *L. calcarifer* and *N. graeffei* move, on average, 4 to 6 km, respectively, from their home location along the main-channel network and hundreds of meters onto the floodplain^43^. We recommend that more targeted research using tagging techniques is needed to test whether similar movement occurs in the Fitzroy River.

In the Fitzroy River, the base of the food web likely varies with geomorphology and river-floodplain connectivity. For instance, in upland reaches we hypothesise that algal biofilm production is promoted because cobble substrates and shallow water are ideal for algal biofilm growth, and minimal floodplain connectivity reduces the availability of terrestrial carbon. Conversely, non-algal biofilm sources become increasingly important in lowland reaches because unstable sand substrates limit the development of algal biofilms^24,46^, and an established riparian zone and greater river-floodplain connectivity create an abundant and diverse supply of allochthonous carbon^47^. Allochthonous carbon from the floodplain may enter the food web through a detrital pathway or via zooplankton production^21,48,49^. Terrestrial subsidies from the floodplain to the main-channel food web are likely to vary from year to year and should be most evident after large wet season flows. Targeted research is needed to test these predictions and assess the importance of floodplain leaf litter in the lowland refuge pools of this river.

### Conclusions and implications for water management

This study adds to our knowledge of energetics of the Fitzroy River. There is now considerable evidence that algal biofilm carbon underpins fish production in upland main-channel and tributary sites^16,17^, on the floodplain (this study), and for large-bodied fish (*L. calcarifer*, *N. graeffei*) in lowland main-channel pools (this study). Given that groundwater upwelling promotes the development of algal biofilms in the main-channel^24^ we recommended that water extraction has a minimal impact on surface–groundwater interactions. Importantly, this study found that non-algal biofilm sources of energy, i.e. leaf litter or phytoplankton, sustain a considerable proportion of fish biomass in lowland pools during the dry season. These pools are refuges in this intermittent system, and it is critical that their food web is not impacted by water resource development. Given the importance of riparian vegetation in the supply of carbon to main-channel dry-season food webs, management policies should also aim to protect the surface and sub-surface flows that sustain riparian vegetation. We found no direct evidence that remote carbon sources, from either the floodplain or estuary, deliver energy to fish in lowland main-channel pools. Additional research using different methods (e.g. tagging), examining greater numbers of fish (investigating estuarine subsidy), and sampling the floodplain and main-channel concurrently is needed, as are food web studies during periods of very high and very low flows. Until this occurs, we encourage managers to take a precautionary approach to flow policy.

## Methods

### Use of animals

This research was carried out under Fisheries exemption #2974 and The University of Western Australia’s Animal Ethics permit RA/3/100/1536 (Animal Ethics Committee, The University of Western Australia). All researchers conducting field work had a valid Permission to Use Animals (PUA) licence to use animals for scientific purposes as per the Animal Welfare Act 2002 (Western Australia). All field procedures were performed in accordance with relevant guidelines and regulations.

### Study area

The Fitzroy River drains a savannah landscape of the wet-dry tropics of northern Australia. The rivers’ flow regime is categorised as predictable summer highly intermittent (class 10, Kennard et al.^50^). Wet season occurrence is predictable (Nov–Mar) but varies in magnitude from year to year^10^. Strong seasonal and inter-annual flow variation create periods of extremely high and very low water availability in the catchment. Our study occurred in the lower reaches of the river; the section considered most likely to be impacted by future water extraction. The main-channel in this section displays a sinuous planform and contains alternating deep and shallow reaches^51^. The floodplain is dominated by cracking clays and dissected by distributary creeks that distribute water from the main-channel during elevated within-bank flows^51^.

### Approach

Our methods proceed in two main steps; first, we describe the food web for two habitats within the lowland area of Fitzroy River; main-channel pools and the floodplain. We used two natural tracers, stable isotopes of carbon (^13^C/^12^C) and nitrogen (^15^N/^14^N) and applied a Bayesian mixing model approach to determine the sources of energy supporting fish species present in each habitat type. Second, we applied multiple lines of reasoning to investigate evidence that energy in large-bodied fish in main-channel pools may have been acquired remotely, i.e. from the floodplain or from the estuary. To this end, we used stable isotopes of sulphur (^34^S/^32^S) to investigate evidence of marine residency in a subsample (n = 3) of large-bodied migratory fish (*L. calcarifer*, *N. graeffei*) and a control non-migratory freshwater species (*H. jenkinsi*). Marine fish typically display δ^34^S values from 13 to 18‰, freshwater fish display values between − 8 and + 8‰, and fish that feed and move between these habitats typically display intermediate isotopic sulphur signals^31,52,53^.

### Data sets

We used two data sets, one collected in the main river channel and one collected on the floodplain, augmented with fish collected opportunistically from the estuary. The main-channel data set was collected from three physically similar pools in the lowland river during the dry season (October 2017) (Supplementary Figs. S1, S2). These sites were between 23 and 65 km upstream of the estuary. All sites had steep banks and were overhung by riparian vegetation consisting primarily of paperbarks (*Melaleuca argentea* and *M. leucadendra*) and freshwater mangrove (*Barringtonia acutangula*). When sampled, pools had little to no flow and were 1.5, 1.9 and 2.5 km long, ~ 60 m wide and 0.5 to 3.5 m deep. The floodplain data set was collected from two flood-runner creeks and two floodplain proper wetlands (see Figs. S1, S2, Supplementary Information) during the wet season (March 2018) (see Figs. S1, S2) The estuarine data set was limited to the October 2018 collection of muscle tissue from three *L. calcarifer* from King Sound (the large marine embayment into which the Fitzroy River debouches), which provided a sulphur (^34^S/^32^S) reference value for the marine environment.

### Food web sample collection

The main-channel data set included comprehensive sampling of all sources and consumers (macroinvertebrates and fish). The floodplain data set only included samples of the two dominant sources of primary production (algal biofilm and leaves) and fish consumers (i.e. macroinvertebrates weren’t sampled).

#### Source material

Terrestrial/riparian leaves (e.g. *Melaleuca*, *Barringtonia*, *Eucalyptus* spp., *Acacia* spp.) were abscised and in various states of decomposition. Coarse particulate organic matter (CPOM) was collected with a 1 mm sweep net. Algal biofilms (periphyton) were collected in main-channel pools by scraping the surface of logs using a toothbrush or razor. Algal biofilms in floodplain habitats were collected from emergent vegetation or cobbles using a toothbrush. Biofilm collections were filtered through a quartz 47 µm filter paper. Aquatic macrophytes (*Chara, Valisneria*), filamentous alga (*Spirogyra*) and blue green algae were collected by hand from main-channel pools. Seston, which is a combination of organismal, i.e. phytoplankton and zooplankton, and nonliving matter, i.e. fine organic matter, floating in the water column was collected from the middle of main-channel pools using a multi-tiered plankton net (500 µm metal sieve at mouth, 250 µm cloth net within a 53 µm net) towed for 300–600 m from a boat during extremely clear water conditions (turbidity < 1.0 NTU). Two seston size fractions were thus collected: a 250–500 µm size fraction, considered to represent the zooplankton component and a 53–250 µm fraction considered to represent the phytoplankton component. Our results indicated that the fine fraction had a δ^13^C of − 30.8 which aligns well with zooplankton isotope values recorded elsewhere in Australia (mean − 31.1^54^). The δ^15^N value of the fine seston fraction was 1.53 lower than the coarse fraction which also broadly aligns with trophic enrichment noted between phytoplankton and zooplankton reported elsewhere in the world^55,56^. Fine particulate organic matter (FPOM) was collected from the pool bottom using a 250 µm sweep net. All sources of primary production were frozen in the field.

#### Consumers

Fish were sampled using a combination of gears including seine, gill and cast nets, hook and line and box traps. Macroinvertebrates (molluscs, insects and macro-crustaceans) were collected using a combination of dip netting (250 µm net), light traps or by hand. All macroinvertebrate and fish samples were frozen soon after collection.

### Laboratory processing and isotope analysis

Sources and consumer material were cleaned of contaminants. Invertebrates were picked from FPOM samples, and FPOM removed from filamentous algae samples. Macrophytes were washed in distilled water and gently rubbed to remove epiphytic alga. Macroinvertebrates were identified under a dissecting microscope and sorted to family where possible. Tissue from bivalve molluscs and small shrimp was removed from their shells, but microcrustaceans (e.g. Cladocera) were included whole. Due to the small size of most macroinvertebrates, the majority of samples were composed of numerous individuals. Fish were dissected and a portion of their white lateral dorsal muscle excised. δ^13^C values of white muscle are less variable than for other tissue types, hence more suitable for ecological analysis^57^. Samples were freeze-dried at − 60 °C for a minimum of 48 h and ground to a fine powder by ball bearings or pestle and mortar. Samples were weighed (to four decimal places) and placed into tin capsules for analysis. Typically, between 1.0 to 1.1 mg of plant matter between 0.5 to 0.6 mg of animal matter was used for stable isotopic analysis.

Samples were analyzed using a continuous flow system consisting of a Delta V Plus mass spectrometer connected to an elemental analyzer Thermo Flush 1,112 via Conflo IV (Thermo-Finnigan/Germany). Samples were analysed concomitantly for nitrogen and carbon, but separately for sulphur. Stable nitrogen, carbon and sulphur isotope compositions are reported using standard δ-notation, see Eq. (1)^58^, after multi-point normalization of raw isotopic data to isotope international reference scale. For the normalization procedure international standards provided by International Atomic Energy Agency (δ^13^C—NBS22, USGS24, NBS19, LSVEC; δ^15^N—N1, N2, USGS32; δ^34^S—VCDT) and laboratory standards were used^58^. The uncertainty associated with the stable isotope analyses (1σ—standard deviation) was not more than 0.10‰.

1 $$\mathrm{\delta X}=\left[\frac{Rsample}{Rstandard}\right]-1 x {10}^{3}$$

A subset of fish muscle tissues displayed high C:N ratios (> 3.5) indicating they contained a high lipid content, which can bias analyses^59^. To counter this potential bias, we corrected for lipid content using the Fry (FMB) method, see Eq. (2)^60^.

2 $$lipid \, corrected\, {\updelta }^{13}\mathrm{C}=uncorrected \, {\updelta }^{13}\mathrm{C}+6- \frac{(22.2)}{C:N ratio}$$

### Data analysis

We used isotope biplots to visually represent the food web and used the mixing model mixSIAR^30^ to determine the relative contribution of different carbon sources to the diet of different fish species collected in lowland main-channel pools and floodplain habitats. The approach uses a Bayesian model and a Gaussian likelihood to estimate the proportion of different food sources to sustain the diet (isotopic value of muscle tissue) of fish consumers. The method is considered superior to other mixing models (e.g. IsoSource) because it is better able to incorporate uncertainty and variation^61^ and allows the inclusion of fixed and random effects^30^.

Our approach to the analysis was to create a single global model structure that contained three covariates, including species as a random effect, site as a random effect, and fish length as a fixed effect and model them on the isometric log-ratio scale (ILR) as:

3 $$\mathrm{ILR}\left({\updelta }^{13}\mathrm{C}\right)=\mathrm{intercept}+\mathrm{species}[\mathrm{R}]+\mathrm{site}[\mathrm{R}]+\mathrm{length}$$

where ${\updelta }^{13}\mathrm{C}$ is the concentration of the carbon isotope extracted from each fish’s tissue sample. We assumed that the effects of species on diet proportions were random effects across species to reduce the probability of spurious results and increase statistical power for species with sparse data^62^. We assumed site as a random effect to account for variation in diet proportions among sites and reduce the likelihood for pseudo replication due to multiple samples collected within sites. Standard length for each fish species was centred on zero and divided by a common standard deviation. This scaling formalizes the assumption that length does not affect the diet proportions across species but has the potential to influence diet proportion across lengths within species.

We applied this global model to three different fish feeding guilds (i.e. omnivores, herbivores, and carnivores) and two different habitats (i.e. main-channel and floodplain) for a total of five independent analyses. Independent analyses had to be run for each feeding guild because the mixSIAR R package only allows the input of one trophic enrichment factor for each isotope (C, N) and the inclusion of fish from different trophic levels (different δ^15^N values) within the same analysis would generate spurious results^63^. We considered all possible combinations of our covariates as plausible models (total of 8 competing models per feeding guild/habitat combination) and used leave-one-out cross validation (LOO) weights to evaluate support for different models and model average diet proportions. We model-averaged parameter estimates by calculating a weighted average of posterior distributions of diet proportions for each competing model (using LOO model weights). We report results as the proportion of algal biofilm in the diet, but strictly speaking it is the proportion of muscle tissue that contains carbon produced originally by algal biofilm production. We don’t report the proportion from non-algal biofilm sources because our analysis only included two sources, hence the proportion of non-algal biofilm sources is merely one minus the proportion of local algal biofilm. Justification of the source and trophic enrichment factor inputs into the mixSIAR model is provided below.

#### Source inputs

Source data were pooled across sites to increase sample size as too few replicates were collected at each site to allow investigation of among-site variation in sources. Some potential sources were excluded from the analysis because *a-priori* information indicates that they are not assimilated into the consumer food web and because the mixing model performs best with fewer sources^61^. Macrophytes (*Chara*, *Valisneria*) were excluded because their carbon is thought to support a microbial loop rather than be incorporated into higher order animals see review by^35^. Blue green algae and filamentous macroalgae *Spirogyra* were excluded because studies in northern Australia have revealed that their carbon is rarely incorporated into metazoan production^32^. In main-channel pools, preliminary appraisal of sources in two-dimensional isospace revealed considerable overlap between the fine fraction of seston and CPOM (r > 0.8) indicating that the model would struggle to separate them. For this reason, data from these sources were pooled prior to analysis and was considered as a joint phytoplankton/leaf litter pathway. Two carbon sources were included in the mixSIAR model for main-channel and floodplain data sets. The first was algal biofilm, the second was non-algal biofilm carbon which was CPOM (leaf litter) for the floodplain 2018 data set and the combination of CPOM and seston 53–250 μm for the main-channel dataset. Mean values and standard deviations input into mixSIAR are presented in Table 1. We stress that our mixing models do not identify actual fish diet, rather they reveal which of the two alternate energetic pathways, i.e. algal biofilm or non-algal biofilm (e.g. leaf litter/phytoplankton), fish are most reliant on.

#### Trophic enrichment factor inputs

The mixing model relies on overlap between consumer and source values within a two-dimensional *iso-space*^64^. To ensure overlap, source values need to be adjusted for isotopic fractionation that occurs across trophic levels. Thus, the model requires inputs of trophic enrichment factors (TEF) for δ^13^C and δ^15^N. As per Blanchette et al.^65^ we used regional δ^15^N TEF’s for nitrogen based on empirical data^66^ from food web studies in Australian dry tropical rivers: 3.9 ± 1.3 (SD) for herbivorous/detritivorous fish and 5.7 ± 1.7 (SD) for carnivorous fish. We used a mid-way TEF for omnivorous fish 4.8 ± 1.7 (SD). The trophic group membership of fish was determined using the literature^67^. Species considered herbivores/detritivores included *N. erebi*, *P. ordensis*, and *Selenotoca multifasciata*). Species considered carnivores included *Strongylura krefftii*, *Elops hawaiiensis*, and *L. calcarifer*. All other species were considered omnivores. For carbon, we used the widely adopted δ^13^C TEF value of 0·4 ± 1·3‰ for each trophic level; hence 0.4 for herbivores, 0.8 for carnivores, and 0.6 for omnivores which are a mixture of both trophic levels^68^. Prior to running the SIAR analysis we constructed *iso-space* plots to confirm that source and consumer values were overlapping^64^. Models were run with 200,000 iterations and a 50,000 burn in, and priors were set as uninformed. We used Gelman-Rubin Diagnostics to assess model fit. All statistical procedures were performed using the statistical software R (R Project for Statistical Computing, Vienna, Austria).

To investigate evidence of marine residency we used non-parametric Kruskal–Wallis rank sum tests, which was appropriate given the small sample size of the data sets. To examine floodplain to main-channel subsidies we checked if algal biofilm δ^13^C values differed between floodplain and main-channel biofilms. To examine estuary to main-channel subsidies were checked if δ^34^S varied between large-bodied migratory species *L. calcarifer, N. graeffei* and the non-migratory medium bodied species *H. jenkinsi*. Kruskal–Wallis tests were run using the kruskal.test function in R.

## Supplementary information

Supplementary Information.
