# Supplementary material for: New insights into the food web of an Australian tropical river to inform water resource management
Source: Sci Rep. 2020 Aug 31;10:14294. doi: 10.1038/s41598-020-71331-0 (PMC7459293; doi:10.1038/s41598-020-71331-0)
Supplement: Supplementary file 1 — Supplementary Information. [file 41598_2020_71331_MOESM1_ESM.pdf]

Supplementary Material

New insights into the food web of an Australian tropical river to inform water resource management

Leah S. Beesley, Bradley J. Pusey, Michael M. Douglas, Daniel C. Gwinn, Caroline A. Canham, Chris S. Keogh, Oliver P. Pratt, Mark J. Kennard, and Samantha A. Setterfield

Table S1. Isotopic values ($\delta^{13}C$ and $\delta^{15}N$, ‰) and nutrient makeup (C%, N% and C:N ratio by weight) of sources of primary production supporting the dry season aquatic food web in the lowland Fitzroy River main-channel (October 2017). Samples were pooled across the three sampling localities.

	n	— $\delta^{13}C$ —		—$\delta^{15}N$ —		C%	N%	C:N
Food Source		mean	s.d	mean	s.d	mean	Mean	mean
Algal biofilm	4	-23.5	2.42	1.77	1.48	17.5	0.57	30.7
Spirogyra	2	-29.7	0.38	0.28	0.67	28.0	1.87	14.0
Blue-green algae	1	-30.0	-	0.06	-	15.7	1.74	9.0
Seston 53-250 µm	5	-30.8	0.49	3.20	1.85	-	-	19.8
Seston 250-500 µm	4	-30.8	0.54	4.73	1.33	-	-	51.9
Macrophyte	4	-21.5	1.44	4.09	2.10	27.2	0.98	41.5
FPOM	4	-29.2	1.25	2.59	0.77	18.8	0.60	28.6
CPOM	4	-31.3	1.45	1.81	1.46	40.3	0.71	94.9
Terrestrial leaves	10	-32.3	0.89	2.61	0.88	49.5	0.39	364:1

Table S2. Isotopic values ($\delta^{13}C$ and $\delta^{15}N$, ‰) of macroinvertebrate functional feeding groups (FFG) and families from lowland main-channel pools in the late dry season (October 2017). Data were pooled for the three study locations.

FFG	Order or Family	No. of replicates	— $\delta^{13}C$ — mean	s.d.	—$\delta^{15}N$ — mean	s.d.
Filterer	Hyriidae	3	-35.1	1.444	6.2	0.399
Filterer	Calanoidea	2	-33.1	0.608	5.0	0.076
Grazer	Tridactylidae	1	-22.7	NA	2.3	NA
Scraper	Baetidae	3	-32.3	0.672	3.9	1.472
Scraper	Caenidae	3	-31.6	1.536	3.2	1.117
Predator	Ceratopogonidae	2	-31.1	0.095	7.0	1.006
Predator	Coenagrionidae	3	-30.3	0.655	5.6	0.807
Predator	Gerridae	1	-29.6	NA	4.9	NA
Predator	Gomphidae	1	-31.4	NA	5.3	NA
Predator	Gyrinidae	1	-35.4	NA	7.7	NA
Predator	Libellulidae	2	-30.3	0.412	5.0	0.347
Predator	Micronectidae	2	-32.1	2.675	5.2	0.329
Predator	Notonectidae	1	-29.4	NA	5.7	NA
Predator	Pleidae	1	-24.5	NA	3.8	NA
Multiple	Chironomidae	1	-34.2	NA	8.2	NA
Multiple	Culicidae	1	-33.3	NA	4.2	NA
Multiple	Leptoceridae	1	-30.0	NA	6.1	NA

Table S3. The average size of fish (standard length, mm) and freshwater prawns (carapace length, mm) collected from lowland main-channel pools in the late dry season (October 2017) and floodplain sites during the late wet season (March 2018). Data were pooled across study sites within each habitat category. * denotes the freshwater prawn. “-“ represents none collected. Sample size (n) and the range in length are also provided.

**Species name**	**Common name**	**Main-channel Pools**	**Floodplain**
		Mean (Range), n	Mean (Range), n
Ambassis spp.	Glassfish	-	23 (14-34) n=11
Anodontiglanis dahli	Toothless catfish	-	64, n=1
Elops hawaiensis	Hawaiian giant herring	348 (260-437) n=2	-
Gerres filamentosus	Threadfin silver biddy	88 (81-95) n=2	-
Hannia greenwayi	Greenway's grunter	102 (89-117), n=5	-
Hephaestus jenkinsi	Western sooty grunter	142 (100-203), n=6	-
Lates calcarifer	Barramundi	301 (236-382), n=15	-
Leiopotherapon unicolor	Spangled perch	108 (99-116), n=3	56 (29-98) n=22
*Macrobrachium spinipes**	Cherabin	23 (7-57), n=30	25(14-44) n=6
Melanotaenia australis	Western rainbowfish	-	35 (17-51) n=11
Nematalosa erebi	Bony bream	139 (98-190), n=12	48 (19-89) n=10
Neoarius graeffei	Blue catfish	274 (147-374), n=16	173 (168-178) n=2
Neosilurus ater	Black catfish	-	94(70-118) n=2
Neosilurus hyrtlii	Hyrtl's catfish	-	68 (54-122) n=6
Planiliza ordensis	Diamond mullet	142 (98-172), n=8	-
Selenotoca multifasciata	Banded scat	109 (82-137), n=6	-
Strongylura krefftii	Freshwater longtom	272, n=1	-
Toxotes kimberleyensis	Kimberley archerfish	94 (59-125), n=13	25 (18-29) n=5

Table S4. Estimated reliance on local algal biofilm carbon from mixing models (SIAR) using 13C and 15N for fish in floodplain habitats during the wet season. Tables show mean, standard deviation (s.d.) and percentiles (0.025, 0.25, 0.75, 0.975). Species codes are as per Fig 1.

Species	Mean	s.d.	0.025	0.25	0.75	0.975
A.dah	0.587	0.207	0.235	0.427	0.739	0.987
Amb	0.903	0.081	0.712	0.855	0.97	1
E.haw	-	-	-	-	-	-
G.fil	-	-	-	-	-	-
H.gre	-	-	-	-	-	-
H.jen	-	-	-	-	-	-
L.cal	-	-	-	-	-	-
L.uni	0.908	0.075	0.735	0.859	0.97	1
M.aus	0.766	0.105	0.571	0.693	0.833	0.988
M.spi	0.706	0.114	0.506	0.625	0.775	0.97
N.ate	0.682	0.173	0.382	0.55	0.814	0.996
N.ere	0.368	0.086	0.208	0.313	0.416	0.551
N.gra	0.815	0.131	0.535	0.723	0.926	0.999
N.hyr	0.514	0.094	0.355	0.45	0.57	0.722
P.ord	-	-	-	-	-	-
S.kre	-	-	-	-	-	-
S.mul	-	-	-	-	-	-
T.kim	0.688	0.111	0.497	0.609	0.758	0.941

Table S5. Estimated reliance on local algal biofilm carbon from mixing models (SIAR) using 13C and 15N for fish in lowland main-channel pools during the late dry season. Tables show mean, standard deviation (s.d.) and percentiles (0.025, 0.25, 0.75, 0.975). Species codes are as per Fig 1.

Species	Mean	s.d.	0.025	0.25	0.75	0.975
A.dah	-	-	-	-	-	-
Amb	-	-	-	-	-	-
E.haw	0.989	0.037	0.915	0.994	1	1
G.fil	0.039	0.05	0	0.002	0.059	0.175
H.gre	0.053	0.056	0	0.005	0.085	0.197
H.jen	0.026	0.032	0	0.002	0.04	0.111
L.cal	0.994	0.025	0.958	0.997	1	1
L.uni	0.227	0.129	0	0.134	0.32	0.463
M.aus	-	-	-	-	-	-
M.spi	0.319	0.047	0.228	0.289	0.347	0.414
N.ate	-	-	-	-	-	-
N.ere	0.185	0.268	0	0.004	0.239	0.979
N.gra	0.971	0.03	0.89	0.957	0.994	1
N.hyr	-	-	-	-	-	-
P.ord	0.975	0.106	0.671	0.999	1	1
S.kre	0.977	0.07	0.802	0.985	1	1
S.mul	0.01	0.069	0	0	0	0.06
T.kim	0.607	0.068	0.481	0.56	0.649	0.75

Table S6. Estimated reliance on leaf litter or phytoplankton carbon from mixing models (SIAR) using 13C and 15N for fish in floodplain habitats during the wet season. Tables show mean, standard deviation (s.d.) and percentiles (0.025, 0.25, 0.75, 0.975). Species codes are as per Fig 1.

Species	Mean	s.d.	0.025	0.25	0.75	0.975
A.dah	0.413	0.793	0.765	0.573	0.261	0.013
Amb	0.097	0.919	0.288	0.145	0.03	0
E.haw	-	-	-	-	-	-
G.fil	-	-	-	-	-	-
H.gre	-	-	-	-	-	-
H.jen	-	-	-	-	-	-
L.cal	-	-	-	-	-	-
L.uni	0.092	0.925	0.265	0.141	0.03	0
M.aus	0.234	0.895	0.429	0.307	0.167	0.012
M.spi	0.294	0.886	0.494	0.375	0.225	0.03
N.ate	0.318	0.827	0.618	0.45	0.186	0.004
N.ere	0.632	0.914	0.792	0.687	0.584	0.449
N.gra	0.185	0.869	0.465	0.277	0.074	0.001
N.hyr	0.486	0.906	0.645	0.55	0.43	0.278
P.ord	-	-	-	-	-	-
S.kre	-	-	-	-	-	-
S.mul	-	-	-	-	-	-
T.kim	0.312	0.889	0.503	0.391	0.242	0.059

Table S7. Estimated reliance on leaf litter or phytoplankton carbon from mixing models (SIAR) using 13C and 15N for fish in lowland main-channel pools during the dry season. Tables show mean, standard deviation (s.d.) and percentiles (0.025, 0.25, 0.75, 0.975). Species codes are as per Fig 1.

Species	Mean	s.d.	0.025	0.25	0.75	0.975
A.dah	-	-	-	-	-	-
Amb	-	-	-	-	-	-
E.haw	0.011	0.963	0.085	0.006	0	0
G.fil	0.961	0.95	1	0.998	0.941	0.825
H.gre	0.947	0.944	1	0.995	0.915	0.803
H.jen	0.974	0.968	1	0.998	0.96	0.889
L.cal	0.006	0.975	0.042	0.003	0	0
L.uni	0.773	0.871	1	0.866	0.68	0.537
M.aus	-	-	-	-	-	-
M.spi	0.681	0.953	0.772	0.711	0.653	0.586
N.ate	-	-	-	-	-	-
N.ere	0.815	0.732	1	0.996	0.761	0.021
N.gra	0.029	0.97	0.11	0.043	0.006	0
N.hyr	-	-	-	-	-	-
P.ord	0.025	0.894	0.329	0.001	0	0
S.kre	0.023	0.93	0.198	0.015	0	0
S.mul	0.99	0.931	1	1	1	0.94
T.kim	0.393	0.932	0.519	0.44	0.351	0.25

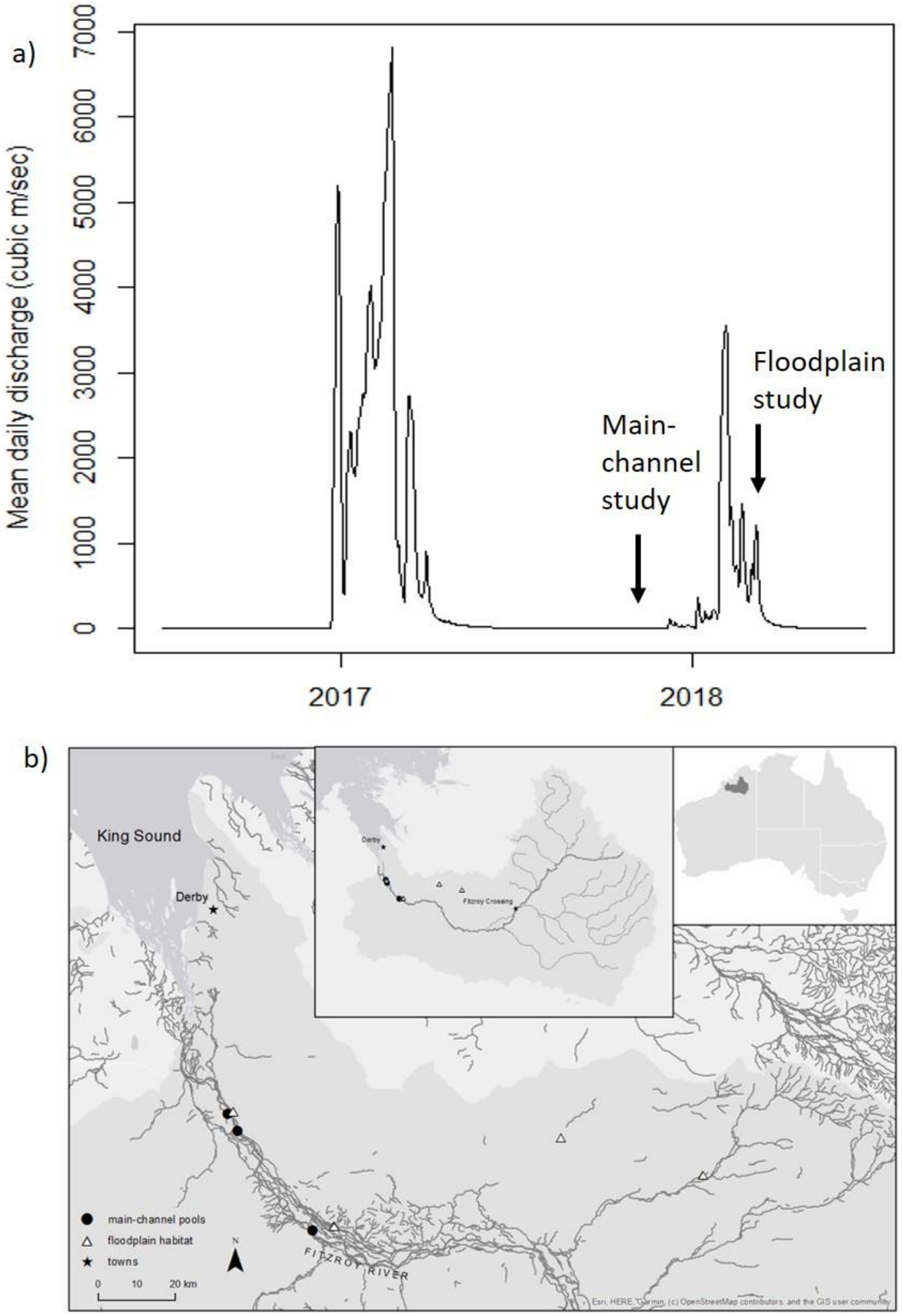

Figure S1. The Fitzroy River showing (a) discharge prior to, and during, the study periods, and (b) the sampling locations for the two freshwater lowland isotope data sets. Main-channel sites (October 2017) shown with black circles and floodplain sites (March 2018) are shown with empty triangles. Panel (b) was generated using ArcGIS version 10.5.1. Redlands, CA: Environmental Systems Research Institute, Inc. https://www.arcgis.com

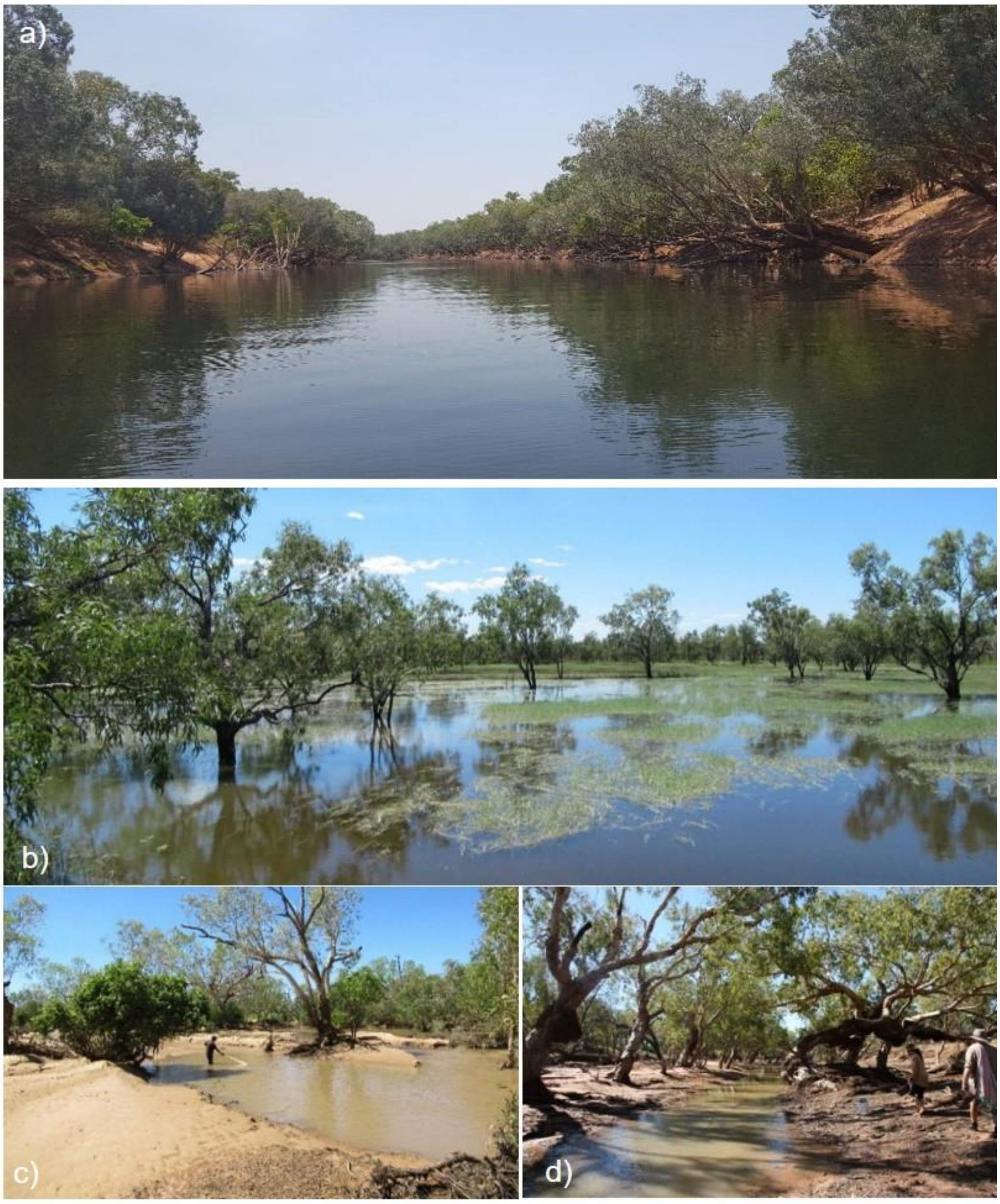

Figure S2. Lowland main-channel pool and floodplain habitats. Panel (a) shows a main-channel pool during the late dry season (October 2017), panels b, c and d show floodplain wetlands and creeks during the wet season (March 2018).
